# Robust photogrammetric scalp morphology estimation for functional optical neuroimaging

**DOI:** 10.1117/1.NPh.12.3.035002

**Published:** 2025-07-28

**Authors:** Abigail L. Magee, Calamity Svoboda, Tessa G. George, Alvin A. Agato, Edward J. Richter, Joseph P. Culver, Adam T. Eggebrecht

**Affiliations:** a Washington University, Department of Biomedical Engineering, St Louis, Missouri, United States; b Washington University, Mallinckrodt Institute of Radiology, School of Medicine, St Louis, Missouri, United States; c Washington University, Department of Electrical & Systems Engineering, St Louis, Missouri, United States; d Washington University, Division of Biology & Biomedical Sciences, St Louis, Missouri, United States; e Washington University, School of Medicine, Department of Imaging Sciences, St Louis, Missouri, United States

**Keywords:** photogrammetry, neuroimaging, functional, optics, high-density diffuse optical tomography, functional near-infrared spectroscopy

## Abstract

**Significance:**

Optical functional neuroimaging relies upon accurate anatomical models to provide optimal data registration and image reconstruction.

**Aim:**

We establish and validate a robust photogrammetric algorithm for scalp morphology that utilizes 3-dimensional (3D) imaging with a photogrammetric cap to provide individualized scalp morphology estimation through hair in the absence of magnetic resonance imaging (MRI).

**Approach:**

The scalp morphology estimation uses a flexible, neoprene cap and 3D-printed photogrammetric modules with fiducials. We create a sparse scalp sampling and align the MNI152 atlas to generate the scalp morphology estimation. We used the international 10 to 20 electroencephalogram positions for alignment and calculated the error as the Euclidean distance among a subspace of modified 10 to 5 electroencephalogram points between surface-based methods and participant-specific MRI.

**Results:**

The scalp morphology estimation error relative to participant-specific MRI had a mean (std) error of 4.27 (2.15) mm, a gold standard volumetric registration of 3.57 (1.69) mm, a four-point scaled atlas of 11.45 (6.00) mm, an unscaled atlas of 5.35 (1.52) mm, and a scalp estimation without cap of 12.42 (6.45) mm. Notably, the scalp morphology estimation demonstrated lower variance in the spatial distribution of error, indicating robustness to idiosyncratic head shapes.

**Conclusions:**

Our scalp morphology estimation algorithm is robust in the presence of hair, provides accurate participant-specific head shapes without requiring MRI, and scales to other cap and fiducial designs, thus highlighting the utility of this tool for a variety of applications.

## 1 Introduction

Functional magnetic resonance imaging (fMRI) has revolutionized neuroscience as a prime technology for studying human brain function and connectivity.^1^^–^^3^ However, fMRI is infeasible at the critical care bedside, in naturalistic environments, in low-resource settings, or in constraining environments.^1^^–^^3^ Functional near-infrared spectroscopy (fNIRS) measures the changes in near-infrared (NIR) light absorption to estimate changes in a blood oxygenation level–dependent (BOLD) signal in the brain, much like fMRI that measures a BOLD signal via local changes in magnetic susceptibility.^4^^–^^10^ Current commercial and research-grade fNIRS systems have the benefit of portability, wearability, and flexibility for critical care, naturalistic environments, and low-resource settings.^4^^,^^8^^,^^9^^,^^11^^–^^24^ However, a major challenge with fNIRS is the lack of information about the underlying anatomy.^7^^,^^8^^,^^25^^–^^28^ Accurate anatomical information is essential for the precise localization of brain signals both within an individual and across a population.

High-density diffuse optical tomography (HD-DOT) provides fMRI-comparable maps of brain function and connectivity while preserving the benefits of fNIRS imaging systems.^29^^–^^31^ HD-DOT uses dense, overlapping, multidistance measurements to reconstruct three-dimensional (3D) images of the changes in cerebral blood oxygenation.^32^^–^^38^ Compared with other near-infrared neuroimaging techniques, HD-DOT has improved brain specificity and spatial resolution.^26^^,^^27^^,^^35^^,^^37^^,^^39^^–^^41^ However, HD-DOT is a computational imaging technique that requires realistic and accurate models of the head surface size and shape to generate accurate reconstructions of the spatial distributions of the BOLD signal. Participant-specific head models that incorporate structural volumetric information, such as from anatomical MRI and computed tomography (CT), provide optimal reconstructions of spatial maps of brain function with a mean localization error of ∼5  mm.^29^ Unfortunately, MRI and/or CT structural imaging may be unavailable when fMRI is unavailable or untenable. In instances where participant-specific structural imaging is unavailable, atlas-based head models yield reasonable results.^29^^,^^42^ Standard atlases generate accurate results in adults by registering the atlas to the participant’s head surface via cranial or imaging array fiducials.^20^^,^^29^^,^^42^^,^^43^ However, population-based atlases often cannot capture patient-specific idiosyncrasies in head shape, especially the variability present in young children, infants, and underrepresented populations.^20^^,^^29^^,^^42^^,^^44^^–^^46^

Using an atlas model for a participant without any adjustments for size and shape leads to spatial errors in image reconstruction due to the differences in cranial and cerebral brain anatomy between the atlas and the participant.^37^^,^^47^^–^^50^ Accurate spatial registration of a given atlas-based head model to participant anatomy requires structural priors.^30^^,^^42^^,^^46^^–^^49^^,^^51^ However, this method is time-consuming and prone to human measurement error.^43^ An alternative strategy utilizes only four easily identified cranial fiducials (the nasion, the left and right tragus, and the inion).^47^ The four points provide adequate degrees of freedom to scale, rotate, and translate the atlas to approximate the participant’s scalp surface. A common approach for generating such structural priors uses electromagnetically digitized international 10 to 20 electroencephalogram (EEG) positions^52^^,^^53^ to generate an affine transform between an atlas and a participant.^42^ However, in participants whose head surfaces significantly differ from the atlas, this method imparts errors in registration due to the overly sparse registration point set. Photogrammetric algorithms can generate physical measurements using information in images. 3D imaging, or 3D scanning, uses structured light or lasers to generate depth and surface information prior to surface reconstruction.^54^^–^^58^ Photogrammetric algorithms have been successfully implemented for biomedical applications but have not extended to estimating participant-specific scalp surface.^59^^–^^64^

In this study, we establish a strategy for estimating participant-specific scalp surface morphology using a 3D camera and a flexible neoprene-based photogrammetric cap that contains specially designed modules. The modules perform two key functions: (i) the top of each module provides a fiducial that is easily detected by the 3D camera, and (ii) the bottom of the module sits against the scalp surface and, importantly, combs through the participant’s hair. We quantitatively evaluated this strategy of estimating the head size and shape using a direct evaluation against a gold standard of participant-specific anatomical MRI-based head surface morphology in N=10 participants and compared errors to those generated with four common alternatives. Our results show that this method outperformed other methods and show that photogrammetric algorithms must account for hair when estimating the head surface.

## 2 Methods

### 2.1 Participants

We recruited 10 healthy, adult participants (age 21 to 30 years) whose head circumference ranged from 54.5 to 61.4 cm for this study (Table 1). We chose a participant pool with varied hair color, hair thickness, and scalp morphology to establish feasibility across normal population variability. All participants passed MR screening, a questionnaire that identifies contraindications such as ferromagnetic implants or severe claustrophobia, to ensure safe participation and provided informed consent as approved by the Human Research Protection Office at Washington University School of Medicine.

**Table 1:** Participant demographics.

Participant no.	1	2	3	4	5	6	7	8	9	10
Sex	F	M	F	F	M	M	M	M	F	F
Age (years)	23	27	23	32	24	30	26	30	27	27
Head circumference (cm)	56.5	57.5	57.5	54.5	61.4	57.5	60.5	60	57	56.5

### 2.2 Hardware

**Fig. 1:**
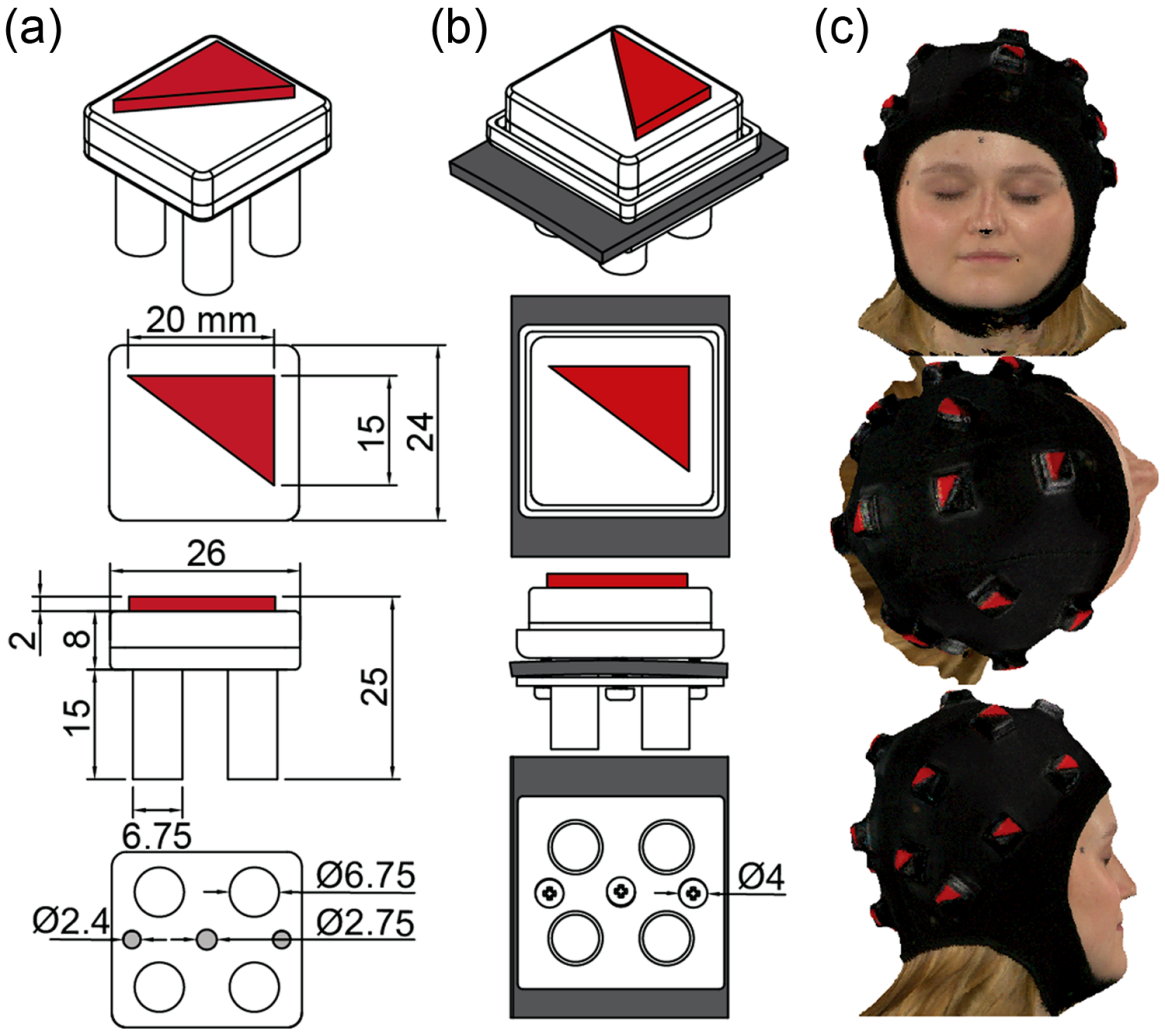
Photogrammetric data collection. (a) Schematic of the photogrammetric module with fiducial triangle in red. All units are in millimeters. (b) Assembled module on neoprene sample (gray). Units for screw diameter are in millimeters. (c) Front view of the 3D scan after importing into MATLAB (top), top-down view of the 3D scan (middle), and right-side view of the 3D scan (bottom).

The scalp surface estimation pipeline incorporated a flexible cap capable of deforming to match the morphology of participants’ scalps with high-contrast fiducials that traditional two-dimensional (2D) or 3D cameras easily identify (Fig. 1). We constructed the cap base from neoprene for its ability to conform to participant-specific head circumferences and shapes while maintaining placement both on the participant and the module on the scalp. In addition, the neoprene cap allowed individual manipulation of the modules to comb through the hair and ensure contact with the scalp. The fiducial modules were 3D printed black cubes with red 3 to 4 to 5 right triangle fiducials and affixed to the neoprene using a 3D printed tray and nylon screws. The bright red triangle fiducial provided rotation and orientation of the fiducial relative to the participant and contrasted with the cap. Our fiducial had dimensions of 20 mm for the vertical leg and 15 mm for the horizontal leg of the triangle.

To maintain stability and provide multiple points of contact per module, we designed each rigid module with four, non-deformable feet that were thin enough to comb through the participants’ hair and couple to the scalp while being nonirritating and durable for multiple cap fits. In addition, the modules were of a height (25 mm) that fit into a standard MRI head coil to allow for direct MRI-based validation of the module locations. We placed 17 photogrammetric modules distributed along the cap surface for a sparse scalp sampling that provided multiple points for registration of the atlas to the participant-specific scalp surface, thereby generating an accurate scalp surface estimation.

### 2.3 Algorithm

**Fig. 2:**
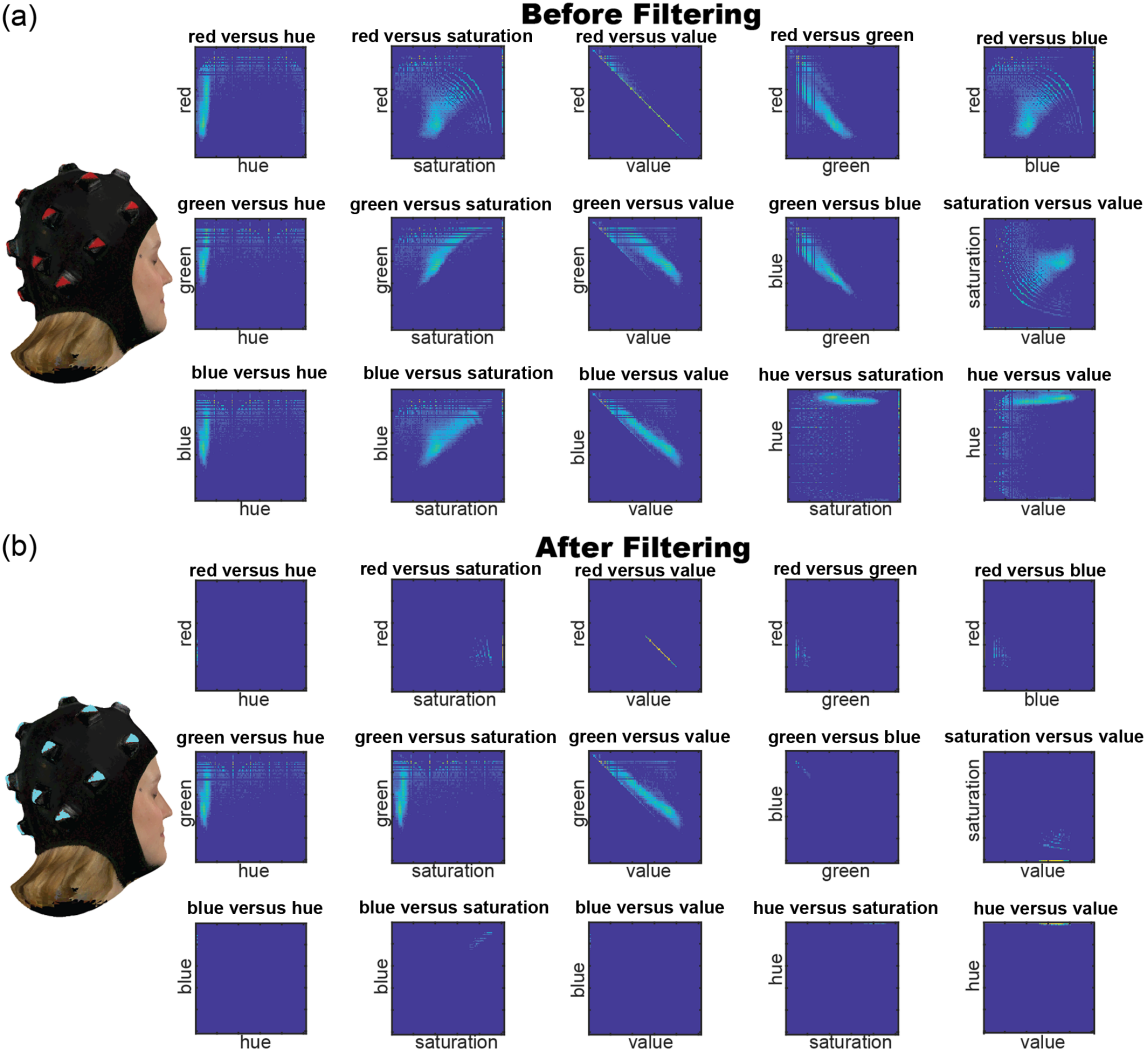
Filtering to isolate fiducials. (a) 3D scan (left) and histograms (right) of red versus blue, red versus green, and hue versus saturation of the mesh (top to bottom) before any filtering has occurred. (b) 3D scan (left) and histograms (right) after filtering.

The 3D camera, Artec LEO,^65^ captured the face, hair, and fiducials of the cap collectively and provided the information necessary to estimate the scalp surface (Fig. 1). To isolate the photogrammetric module fiducials from the surrounding 3D image, the algorithm incorporated filters utilizing normalized color, saturation, and hue values (red≥0.5, saturation≥0.7, and hue≤0.1) (Fig. 2). The saturation filter may vary among participants to compensate for individual differences in skin tone. The algorithm then clusters the filtered nodes into individual fiducials via k-means clustering, an iterative data partitioning algorithm. The filtered nodes were partitioned into 17 clusters given that all nodes within a cluster were within 50 mm of each other, as determined by the number of photogrammetric modules and the physical dimensions of the cap.

The algorithm uses a computer model of the photogrammetric module as the basis for locating the module feet against the scalp. Incorporating a model of the module at this step ensured scalability to other fiducial types and module geometries, as found on currently available commercial and research-grade wearable fNIRS devices.

The algorithm iterates through each cluster to determine the location of the module feet of each individual module through the following sequence. We used principal component analysis to calculate the major axes of the cluster and determine the normal vector that points toward the center of the head. This step provided initial alignment for the model fiducial to the scanned fiducial as well as directional and rotational information for alignment. Next, we rotated the model to align with the scanned module. We save the resulting foot locations from the aligned model as the projected points on the scalp surface.

**Fig. 3:**
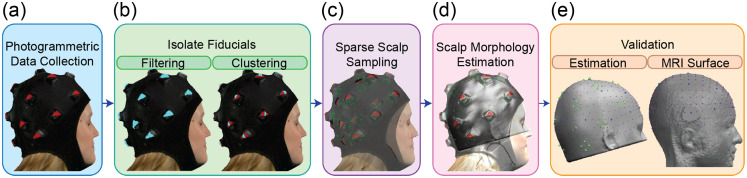
Combination of hardware and algorithm provides robust scalp shape estimation through hair. (a) Participant 3D scan after loading into MATLAB. (b) Nodes that passed color filtering depicted in cyan (left). K-means clustering isolates individual fiducials of filtered points into 17 clusters (centroids of fiducials shown in cyan, right). (c) Sparse scalp sampling (green) overlaid with 3D scan. (d) Scalp morphology estimation using the MNI152 atlas overlaid with the 3D scan and sparse scalp sampling. (e) The international 10 to 20 (blue) and modified 10 to 5 (purple) on the scalp surface estimation with sparse scalp sampling (green, left) and on participant-specific MRI (right).

The module foot locations constituted the sparse scalp sampling used for atlas alignment. We use the MNI152 atlas,^66^ which is an average of 152 T1-weighted MRI scans, because of its high resolution, contrast, and ability to compensate for individual-level variances. We estimated the scale surface by manually aligning the atlas to the sparse scalp sample via rotation, translation, and scaling (Fig. 3).

### 2.4 Validation

We obtained T1-weighted (TR=500  ms, TE=2.9  ms, and $\text{voxel resolution}=1\;{\mathrm{mm}}^{3}$ isotropic) and T2-weighted (TR=3200  ms, TE=564  ms, and $\text{voxel resolution}=1\;{\mathrm{mm}}^{3}$ isotropic) MRI scans with a research-dedicated Siemens 3T Prisma scanner for each participant. We extracted the outer surface of the scalp by segmenting the anatomical MRI volume with normalized thresholding to generate a binary mask of the participant’s head.^29^ Mesh generation proceeded using the binary volume with computational geometry algorithms library (i.e., CGAL) to generate the triangular surface mesh.^67^

We imaged all participants with and without the photogrammetric cap with the Artec LEO 3D scanner immediately following the MRI scan. The 10 to 20 points were localized on both the transformed atlas and participant MRI-derived head surfaces with Mesh2EEG^52^ incorporating the manually selected nasion, inion, and preauricular points. Because the photogrammetric modules are equitably distributed across the scalp, without reference to the 10 to 20 system, the standardized pointset provides an independent method for aligning participant MRI and the estimated scalp surface before validation. Accurate localization of all landmarks: nasion, inion, and preauricular points, is essential as error during localization propagates into estimation of the full set of 10 to 20 points. We mitigated the effect of landmark localization errors using a trained individual to identify landmarks throughout the study. Thus, any errors made by the individual would be consistent and minimize the impact of localization errors on calculated errors. The pipeline employs Mesh2EEG because the algorithm calculates geodesic distances along the surface among landmarks to generate the 10 to 20 pointset. Calculating the geodesic distances ensures individualized international EEG standardized points that reflect idiosyncratic differences in head morphology. We then used a rigid alignment (translation and rotation only) procedure to register the scalp surface estimation and participant-specific head surface measurement using their respective international 10 to 20 points. To quantify the error, we calculated the Euclidean distances among corresponding points within the 10 to 5 montage^52^^,^^53^ that lie above the ears. The adjusted 10 to 5 montage provided error estimation across 289 points around the head.

To contextualize the accuracy of the scalp estimation, we compared the error metrics to those obtained using four other head modeling strategies. First, as a gold standard, we used an affine volumetric registration of the MNI152 atlas to the participant-specific head. We then extracted the head surface of the registered atlas and calculated the error between the registered atlas and the participant head surface as above. Second, we registered the unscaled atlas to the participant-specific scalp via the corresponding 10 to 20 point using a rigid body affine transform (with only translation and rotation). Third, we calculated the affine transform between the MNI152 atlas surface and the participant head surface using only four points at the inion, nasion, and tragus (“basic 4” four-point scaled atlas^43^^,^^47^). Fourth, to explicitly assess the value of taking account of the hair, we estimated the 10 to 20 points on the 3D scan of the participant without wearing the cap and performing scalp morphology estimation without accounting for module leg length. In all cases, after registering with the 10 to 20 points, the Euclidean distances among corresponding non-overlapping 10 to 5 points comprised the error assessment.

### 2.5 Statistical Analyses

The mean, maximum, minimum, and variance of the error for each method were assessed across the 10 to 5 locations within each participant for each registration method. To evaluate registration errors across the participants, we calculated grand means for each method. In addition, to assess registration quality across the head locations, we calculated the mean and variance of error for each 10 to 5 position across participants.

## 3 Results

**Fig. 4:**
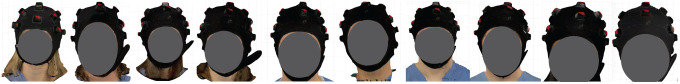
Participant photogrammetric cap placement. Ten healthy, adult participants received MRI and 3D imaging in the photogrammetric cap. The cap placement was nonuniform across the cohort but retained scalp contact with modules in all instances.

We obtained detailed head surface models for N=10 participants (Fig. 4) via participant-specific MRI and atlas-based scalp surface estimation (Fig. 3). The grand mean of the head registration error demonstrates that the scalp surface estimation via the MNI152 atlas approximates the participant-specific head surface to within 5 mm (Table 2). Specifically, the participant-specific head surface produced a mean error of 4.27±2.15  mm for all participants, with a minimum error of 0.65 mm and a maximum error of 12.05 mm. The gold standard approach was the most accurate registration method, with an error of 3.57±1.69  mm, and the scalp surface estimation without the photogrammetric cap was the least accurate, with a 12.42±6.45  mm mean error. Notably, the scalp surface estimation approximates the participant-specific head surface with comparable accuracy to the gold standard volumetric-based affine transform of the atlas to the participant. In addition, this scalp registration procedure outperformed the “basic 4” strategy, the unscaled atlas rigid body registration, and the photogrammetric scalp estimation without the module legs. As expected, registering a participant using a 10 to 20 alignment without accounting for hair performs worse than all other methods.

**Table 2:** Mean values for errors of transforms relative to participant-specific MRI. Values obtained as the mean across all 10 participants.

Transform relative to participant-specific MRI	Mean error (mm)	Minimum error (mm)	Maximum error (mm)	Standard deviation (mm)
Scalp surface estimation	4.27	0.65	12.05	2.15
Volumetric atlas (gold standard)	3.57	0.56	10.07	1.69
“Basic 4” scaled atlas	11.18	0.76	31.85	7.25
Unscaled MNI152 surface	23.79	16.43	31.94	2.98
Scalp surface estimation without cap	12.42	1.85	42.07	6.45
Scalp surface estimation without legs	10.27	4.27	19.61	2.98

**Fig. 5:**
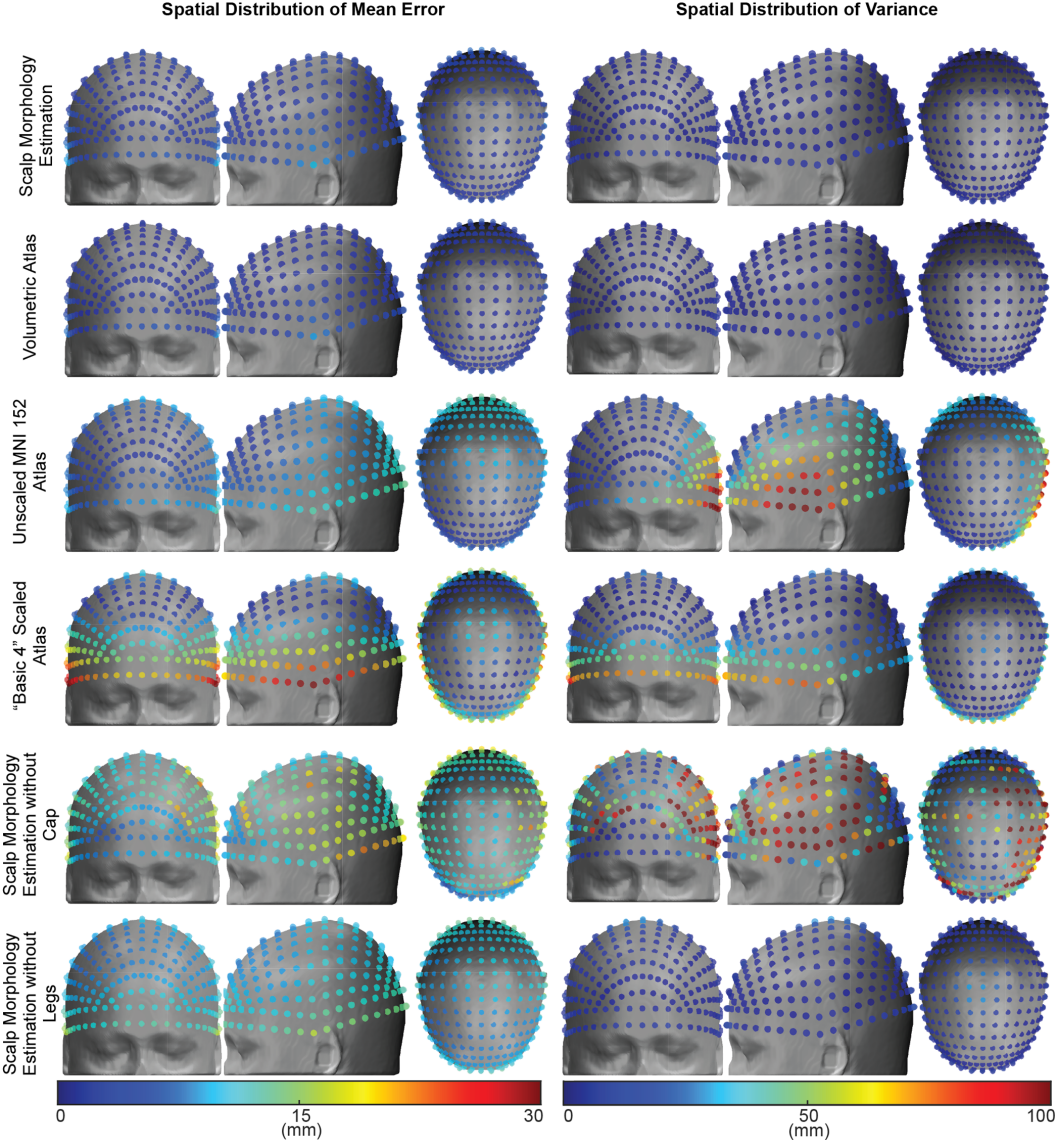
Spatial distribution of mean error and variance. The color of the sphere indicates the mean error (left) or variance (right) in millimeters at that location relative to the participant-specific MRI surface. Top to bottom: scalp morphology estimation, volumetric atlas, “basic 4” scaled atlas, unscaled atlas, scalp morphology estimation without photogrammetric cap, and scalp morphology estimation without photogrammetric module legs.

The spatial distribution of mean and variance of error for each registration method highlights the relative consistency of low registration error across the head surface for both the scalp surface estimated registration and the volumetric registration (Fig. 5). By contrast, the basic 4, unscaled atlas, and scalp surface estimation without the cap exhibit large mean errors, especially on the side of the head, and high variability in error on the side, front, and back of the head. The low variability in our scalp estimation method, as seen in the volumetric registration, highlights that this method manages across idiosyncrasies in head shapes and sizes in our modest population sample far better than other methods.

## 4 Discussion

Accurate HD-DOT reconstruction requires appropriately optimized atlas-based head models for participants whose structural neuroimaging, such as CT or MRI, is unavailable. We proposed photogrammetric hardware and a corresponding algorithm for estimating accurate participant-specific scalp surfaces without requiring anatomical imaging. Our *in vivo* results indicate that this strategy for photogrammetric scalp surface estimation is comparable in accuracy to the current gold standard that is only available when having an actual volumetric representation of the participants’ full head surface, as via MRI or CT. The mean localization error relative to the participant-specific MRI-derived scalp surface is ∼4  mm, which is less than the typical optode separation distance in the vast majority of commercial and known research-grade fNIRS systems.^22^^,^^43^^,^^68^ Previous studies evaluated the accuracy of registration methods for estimating participant-specific scalp morphology and underlying anatomy via optical neuroimaging.^11^^,^^42^^–^^44^^,^^46^^–^^49^^,^^51^^,^^69^^,^^70^ Although most studies focused on analyzing reconstruction rather than participant-specific anatomical imaging, one study compared the accuracy of multiple registration methods in adapting an atlas to participant-specific measurements and achieved mean surface distance errors of 7 mm.^47^ Another study used smartphone-based photogrammetry to register participant optode locations to an MRI atlas^54^ to achieve a median error of 4.5 mm. An additional study investigating scalp surface estimation^71^ achieved a mean reconstruction error of 2.95 mm and a mean registration error of 2.89 mm using 1700-mm path lengths with a neuronavigational tool to scan participant scalp surface. Overall, the atlas-based models achieved good agreement compared with the participant-specific head models; however, the most accurate atlas-based models required time-consuming physical measurements, assuming consistent imaging array placement across participants relative to a phantom, or specialized equipment such as neuronavigational tools.

Previous studies used both linear and nonlinear registration methods to generate atlas-based head models; however, these methods are not robust in the presence of hair and may not be easily scalable to other imaging arrays.^11^^,^^42^^,^^47^^,^^48^^,^^63^^,^^72^^,^^73^ In this study, we developed a photogrammetric algorithm for estimating the scalp surface without physical measurements that is robust in the presence of hair. Our method also achieves a reasonable localization error of 4.27 mm and easily scales to other imaging arrays and fiducial types. Updating the fiducial type or imaging array involves adjusting the color filters and a simplified model to match the required color and geometry. The pipeline does not require further input from the user to proceed with the scalp morphology estimation.

Group analysis of the registration methods revealed greater errors for methods that do not incorporate adequate spatial sampling across the scalp surface (Fig. 5). Conversely, the registration methods with adequate spatial sampling but without compensation for the presence of hair demonstrated more uniform spatial errors but presented mean localization errors larger than those of the proposed method. This disagreement strengthens and highlights the need for accurate atlas-based methods with adequate spatial sampling across the scalp and compensation for hair. Our results support this argument with low localization error due to the level of spatial sampling incorporated in our pipeline and our ability to compensate for the presence of hair. Moreover, the photogrammetric pipeline shows uniform spatial errors that are comparable to the gold standard volumetric affine transform registration of the atlas to participant-specific head surfaces. Finally, the localization errors obtained with the photogrammetric approach compared with the participant-specific head surface are less than those of typical source-detector optode separation, typical gyral span, and mean sulcal width.^74^^,^^75^

A few limitations of the current study warrant discussion. The proposed pipeline uses 3D images of the cap, including the described modules that comb through hair and their fiducials, to obtain the morphological information of the scalp shape and size. Then, the imaging array of choice would be placed on the participant and aligned with the head surface using additional fiducials. The key advancement of our algorithm is that it provides a photogrammetric pipeline to estimate the scalp shape and size through a participant’s hair. While alleviating the need for collecting an anatomical MRI, this pipeline does take roughly 5 min to ensure a comfortable fit of the cap through the hair along with a 3D scan that captures the full perspective of the head. For functional imaging, this cap would then need to be replaced with the fNIRS array of choice. However, if the user is applying an imaging array that contains modules of a known geometry, then the algorithms proposed in this study can be readily applied to estimate the scalp surface using the geometry of the system. Further advancements of this algorithm may provide both the scalp surface and the optode potions of the imaging array. The scalp estimation algorithm is extending to use 2D imaging to provide scalability to the common user and low-resource settings. Our study employed manual alignment for scalp estimation because algorithms such as coherent point drift (CPD) and iterative closest point (ICP) did not align the unmatched point sets used for the scalp surface estimation and do not account for anisotropic scaling, which our pipeline easily incorporates when needed. However, initial alignment using CPD or ICP may be beneficial for other studies and pipelines. In addition, we used adult participants for the study as a proof-of-concept. Future studies will extend the proposed algorithm for use in young children and infants, which are currently underrepresented in atlas-based head models due to structural differences during development, and nonlinear scaling during the registration procedure to improve accuracy.^14^^,^^15^^,^^24^^,^^68^^,^^70^^,^^76^^–^^83^

## 5 Conclusion

Our evaluations indicate that the proposed photogrammetric scalp-surface estimation method provides good localization (∼4  mm) across participants with a variety of head sizes and shapes. Compared with MRI-derived participant head surfaces, the analysis revealed that the mean localization error was within the average source-detector separation distance. In addition, the variability in spatial error across head shapes reveals uniform errors across the surface, which indicates that the pipeline contains adequate spatial sampling for uniformly accurate registration and scalp surface estimation. Our results motivate further application studies that involve 2D imaging and extension to volumetric analyses for optical functional neuroimaging.

## Data Availability

All data and code are available upon request and will be included in a future NeuroDOT update at nitrc.org/projects/neurodot/.
